# Protective effect of empagliflozin against palmitate-induced lipotoxicity through AMPK in H9c2 cells

**DOI:** 10.3389/fphar.2023.1228646

**Published:** 2023-12-05

**Authors:** Min-Woo Song, Wenhao Cui, Chang-Gun Lee, Rihua Cui, Young Ho Son, Young Ha Kim, Yujin Kim, Hae Jin Kim, Sung-E. Choi, Yup Kang, Tae Ho Kim, Ja Young Jeon, Kwan-Woo Lee

**Affiliations:** ^1^ Department of Endocrinology and Metabolism, Ajou University School of Medicine, Suwon, Republic of Korea; ^2^ Department of Hematology, Yanbian University Hospital, Yanji, Jilin, China; ^3^ Department of Biomedical Laboratory Science, College of Software and Digital Healthcare Convergence, Yonsei University MIRAE Campus, Wonju, Republic of Korea; ^4^ Department of Physiology, Ajou University School of Medicine, Suwon, Republic of Korea; ^5^ Division of Endocrinology and Metabolism, Department of Internal Medicine, Seoul Medical Center, Seoul, Republic of Korea

**Keywords:** EMPA, palmitate, cardiomyocyte, AMPK, SGLT2 inhibitor, lipotoxicity

## Abstract

Sodium-glucose cotransporter 2 (SGLT2) inhibitors have recently emerged as novel cardioprotective agents. However, their direct impact on cardiomyocyte injury is yet to be studied. In this work, we investigate the underlying molecular mechanisms of empagliflozin (EMPA), an SGLT2 inhibitor, in mitigating palmitate (PA)-induced cardiomyocyte injury in H9c2 cells. We found that EMPA significantly attenuated PA-induced impairments in insulin sensitivity, ER stress, inflammatory cytokine gene expression, and cellular apoptosis. Additionally, EMPA elevated AMP levels, activated the AMPK pathway, and increased carnitine palmitoyl transferase1 (CPT1) gene expression, which collectively enhanced fatty acid oxidation and reduced stress signals. This study reveals a novel mechanism of EMPA’s protective effects against PA-induced cardiomyocyte injury, providing new therapeutic insights into EMPA as a cardioprotective agent.

## 1 Introduction

Diabetic cardiomyopathy is a severe event in diabetes and obese patients, caused by excessive lipid accumulation in cardiomyocytes (Nakamura and Sadoshima, 2020). The morbidity of cardiomyopathy has recently been rising rapidly, coinciding with the epidemic of diet-induced obesity (Birkenfeld et al., 2019). Elevated free fatty acids (FFAs) in diet-induced obesity and high glucose levels are independently associated with higher risks of cardiac disease (Carpentier, 2018).

Although the heart primarily metabolizes fatty acids, providing 70% of the heart’s energy requirements (Gray and Kim, 2011), excessive free fatty acid delivery to the heart can impair fatty acid oxidation and increase both catabolic and anabolic processes in cardiac tissue, leading to triglyceride accumulation in myocytes (Carley et al., 2004; Buchanan et al., 2005; Herrero et al., 2006). Saturated FFAs like palmitate (PA), compared to unsaturated FFAs, induce the production of cytosolic and mitochondrial reactive oxygen species (ROS) and disrupt Ca2+ homeostasis among the ER, mitochondria, and cytosol, resulting in ER stress, oxidative stress, and subsequent insulin resistance (Wu et al., 2021). Moreover, excessive saturated FFAs and their lipid intermediates can also trigger inflammatory responses (Cohen et al., 2017). Pro-inflammatory cytokines such as TNF-α and IL-1β may directly affect cardiomyocyte injury and heart failure (Hanna and Frangogiannis, 2020; Kaur et al., 2021). Additionally, excessive triglyceride intake induces cellular apoptosis in cardiomyocytes, leading to cardiac tissue dysfunction (Inoue et al., 2013). Therefore, optimizing CPT1 substrate utilization to modulate lipid metabolism is crucial for preventing the progression of diabetic cardiomyopathy.

Sodium-glucose cotransporter 2 (SGLT2) is responsible for glucose absorption (Szekeres et al., 2021). In recent decades, SGLT2 inhibitors like empagliflozin (EMPA) have targeted the renal pathophysiological defects in type 2 diabetes. While SGLT2 inhibitors significantly lower blood glucose, they carry a risk of hypoglycemia as their mechanism of action is independent of insulin and metabolic regulation (Abdul-Ghani et al., 2013; Vallon and Thomson, 2017). However, SGLT2 inhibitors have recently gained attention as novel cardioprotective agents, particularly beneficial in the early stages of heart disease (Zinman et al., 2015; Liu et al., 2021).

Considering that diabetic cardiomyopathy is often asymptomatic in its early stages but can rapidly progress to heart failure, early prevention is vital (Stanton et al., 2021). Despite extensive research on the role of SGLT2 inhibitors in vascular injury, little is known about their direct impact on cardiomyocyte injury *in vitro*. The precise mechanisms by which SGLT2 inhibitors protect against diabetic cardiomyocyte injury through AMPK activation are not fully understood.

In this study, we investigated the mechanisms of EMPA, an SGLT2 inhibitor, on PA-induced diabetic cardiomyocyte injury *in vitro* using H9c2 cells. We found that EMPA protects against excessive PA-induced cardiomyocyte injury, including insulin resistance, inflammatory cytokine production, and apoptosis, through AMPK activation.

## 2 Materials and methods

### 2.1 Reagents

Empagliflozin (EMPA, Cat: HY-108682) and Compound C (Dorsomorphin; Cat HY-13418A) were purchased from MedChemExpress (Monmouth Junction, NJ, United States). Other chemicals, including bovine serum albumin (BSA; 2207008), PA (P5585), and insulin (I9278) were purchased from Sigma-Aldrich (Burlington, MA, United States). 2-[N-(7-nitrobenz-2-oxa-1,3-diazol-4-yl)-amino]-2-deoxyglucose (2*-*NBDG; N13195) was obtained from Thermo Fisher Scientific (Waltham, MA, United States). Cell Death Detection enzyme-linked immunosorbent assay (ELISA^plus^) kit was purchased from Roche Applied Science (Roche Applied Science, Mannheim, Germany). AMP colorimetric assay kit was purchased from Biovision (Cat; K229, Milpitas, CA, United States). Antibodies targeting phospho-AKT (#9271), T-AKT (#9272), phospho-GSK3 α/β (#9331), T-GSK3β (#9315), phospho-JNK (#9251), T-JNK (#9252), phospho-eIF2A (#9721), T-eIF2A (#9722), CHOP (#2895), phospho-NF-κB p65 (#3033), NF-κB p65 (#3034), phospho-p38 (#9211), cleaved caspase-3(#9661), phospho-AMP (#2531), and T-AMP (#2532) were obtained from Cell Signaling Technology (Beverly, MA, United States). Anti-β-actin (A300-491A) antibody was purchased from Bethyl Laboratories (Montgomery, TX, United States).

### 2.2 Preparation of PA

PA/bovine serum albumin (BSA) complex was prepared by soaping PA with sodium hydroxide and mixing it with BSA. Briefly, a 20 mM solution of PA in 0.01 M NaOH was incubated at 70°C for 30 min and the fatty acid soaps were then complexed with 5% fatty acid-free BSA in phosphate-buffered saline (PBS) at a 1:3 volume ratio. The complexed fatty acids consisted of 5 mM PA and 3.75% BSA. The *p*A/BSA conjugates were administered to cultured cells at the indicated concentration of PA.

### 2.3 Cell culture and preparation of tissue samples

Rat cardiomyoblast cell line H9c2 was obtained from American Type Culture Collection (ATCC; Manassas, VA, United States) and maintained in low-glucose (1 g/L) Dulbecco’s modified Eagle’s medium (DMEM) supplemented with 10% fetal bovine serum and antibiotics (100 IU/mL of penicillin and 10 μg/mL of streptomycin) at 37°C in a humidified atmosphere of 5% CO_2_. Rat tissue lysates including liver (RT129), pancreas (RT133), muscle (RT135), heart (RT127), and kidney (RT128) were purchased from Cell Biologics Inc. (Chicago, IL, United States).

### 2.4 Immunoblotting

Cells were lysed with cell lysis buffer [150 mM NaCl, 1% NP-40, 0.5% deoxycholate, 0.1% sodium dodecyl sulfate (SDS), 50 mM Tris–Cl, pH 7.5, proteinase inhibitor and protease inhibitor cocktail (Roche Applied Science, Mannheim, Germany)] in 4°C for 30 min. Whole proteins were extracted by differential centrifugation (10,000 *g*, 10 min), and protein concentrations in lysates were determined using a Bio-Rad Protein Assay Kit (Bio-Rad, Hercules, CA, United States). An equal volume of 2× SDS sample buffer (125 mM Tris–Cl, pH 6.8, 4% SDS, 4% 2-mercaptoethanol, and 20% glycerol) was added to the cell lysates, and equivalent amounts of protein (10 μg) were separated by SDS-PAGE and transferred to polyvinylidene fluoride membranes (Millipore, Bedford, MA, United States). After blocking the membranes with 5% skim milk for 30 min, target proteins were immunoblotted to primary antibodies, followed by secondary antibodies (horseradish peroxidase-conjugated anti-mouse IgG or anti-rabbit IgG). Immunoreactive bands were visualized by enhanced chemiluminescence (Amersham Pharmacia Biotech, Arlington Height, IL, United States).

### 2.5 Quantitative reverse transcriptase-polymerase chain reaction (qRT-PCR)

Total RNA from cells was extracted with RNAiso Plus reagent (Takara Bio, Shiga, Japan). cDNA was synthesized using the AMV reverse transcriptase and random 9-mers supplied with the TaKaRa RNA PCR Kit (version 3.0; TaKaRa Bio, Shiga, Japan). The primer sets for PCR amplification are listed in Supplementary Table S1. qRT-PCR was performed with SYBR Green (TaKaRa Bio, Shiga, Japan) using a TaKaRa TP-815 instrument. Relative quantities of amplified DNA were analyzed using the software bundled with the TP-815 instrument and normalized to mouse RPL32 mRNA levels.

### 2.6 2-NBDG uptake assay

Heart-derived H9c2 cells were treated with or without PA for 8 h and starved for 4 h. Cells were then incubated in Krebs-Ringer bicarbonate buffer (pH 7.4) containing 2% BSA at 37°C for 30 min and then treated with 500 μM 2-NBDG with or without 100 nM insulin at 37°C for 2 h. Collected cells were lysed with cell lysis buffer and centrifuged at 12,000 rpm for 30 min. The fluorescence intensity of 2-NBDG in the separated supernatant was measured (excitation: 475 nM; emission: 550 nM) using a SpectraMax iD3 Fluorescence microplate reader (Molecular Devices, Sunnyvale, CA, United States).

### 2.7 Analysis of oxygen consumption rate (OCR)

H9c2 cells were plated into XF24 cell culture microplates and cells were either treated or not treated with drugs, depending on the experimental condition. Following treatment, the H9c2 cells were pre-washed with Krebs-Ringer bicarbonate (KRB) buffer and equilibrated with XF assay medium supplemented with 2.5 mM glucose/50 mM carnitine/0.2 mM PA (for PA OCR) at 37°C in a CO_2_-free incubator for 1 h. The OCR of PA as a carbon substrate was measured using an XF24 extracellular analyzer (Seahorse Bioscience, North Billerica, MA, United States).

### 2.8 Measurement of AMP

AMP level was determined using an AMP colorimetric assay kit (Biovision, Milpitas, CA, United States). Briefly, cells were scraped, washed with phosphate-buffered saline (PBS), and homogenized in cold assay buffer supplied by the kit. Supernatant after differential centrifugation (10,000 g, 10 min) was collected and used to measure AMP level. Samples (2–20 ul) were added into a 96-well plate and sample reaction mix supplied by the kit was then added to each test sample. Enzymatic reaction was performed at 37°C for 60 min. Absorbance was measured at 570 nm. AMP level was determined regarding the AMP standard curve.

### 2.9 DNA fragmentation assay

Cell death was determined by measuring fragmented DNAs using Cell Death Detection enzyme-linked immunosorbent assay (ELISA^plus^) kit (Roche Applied Science, Mannheim, Germany). Briefly, cells were lysed with lysis buffer supplied with the kit. After centrifugation (200 g, 10 min), the supernatant was transferred to an anti-streptavidin-coated microplate. Anti-DNA monoclonal antibody conjugated with peroxidase (anti-DNA-POD) and anti-histone-biotin was added. After incubation at 25°C for 90 min, wells were rinsed with incubation buffer (supplied by the kit) three times. Color was developed by adding 2, 20-azino-di-[3-ethylbenzthiazoline sulphonate] (ABTS) substrate solution, followed by incubation with shaking at 250 rpm for 10–20 min. The amount of peroxidase retained in the nucleosome complex was determined by measuring the absorbance value at 405 nm on a microplate reader.

### 2.10 Statistical analysis

All experiments were repeated at least three times. All data are expressed as the mean ± standard error of the mean (SEM) and were analyzed using GraphPad Prism 9.2.0 (GraphPad Software Inc., San Diego, CA, United States). Statistical analysis was performed via one-way analysis of variance (ANOVA) with the Bonferroni *post hoc* test. A probability (*p*) value less than 0.05 (*p* < 0.05) was considered statistically significant.

## 3 Results

### 3.1 SGLT2 is expressed in cardiomyocyte

To investigate the impact of SGLT2 inhibitors on cardiomyocytes, we first assessed both mRNA and protein expression levels of SGLT2 in different rat tissues. We found that mRNA expression of SGLT2 is present in cardiac tissues, although at levels much lower than in the kidney and pancreas, but higher than in the liver and muscle (Supplementary Figure S1A). Conversely, protein expression levels of SGLT2 were similar across several tissues (Supplementary Figure S1C). Additionally, we observed mRNA and protein expression of SGLT2 in the H9c2 cardiomyocyte cell line and the FaO hepatoma cell line (Supplementary Figures S1B, D). This suggests that the effects of SGLT2 inhibitors on cardiomyocytes extend beyond their role in glycosuria.

### 3.2 Treatment of PA-evoked cardiomyocytes injury

**FIGURE 1:**
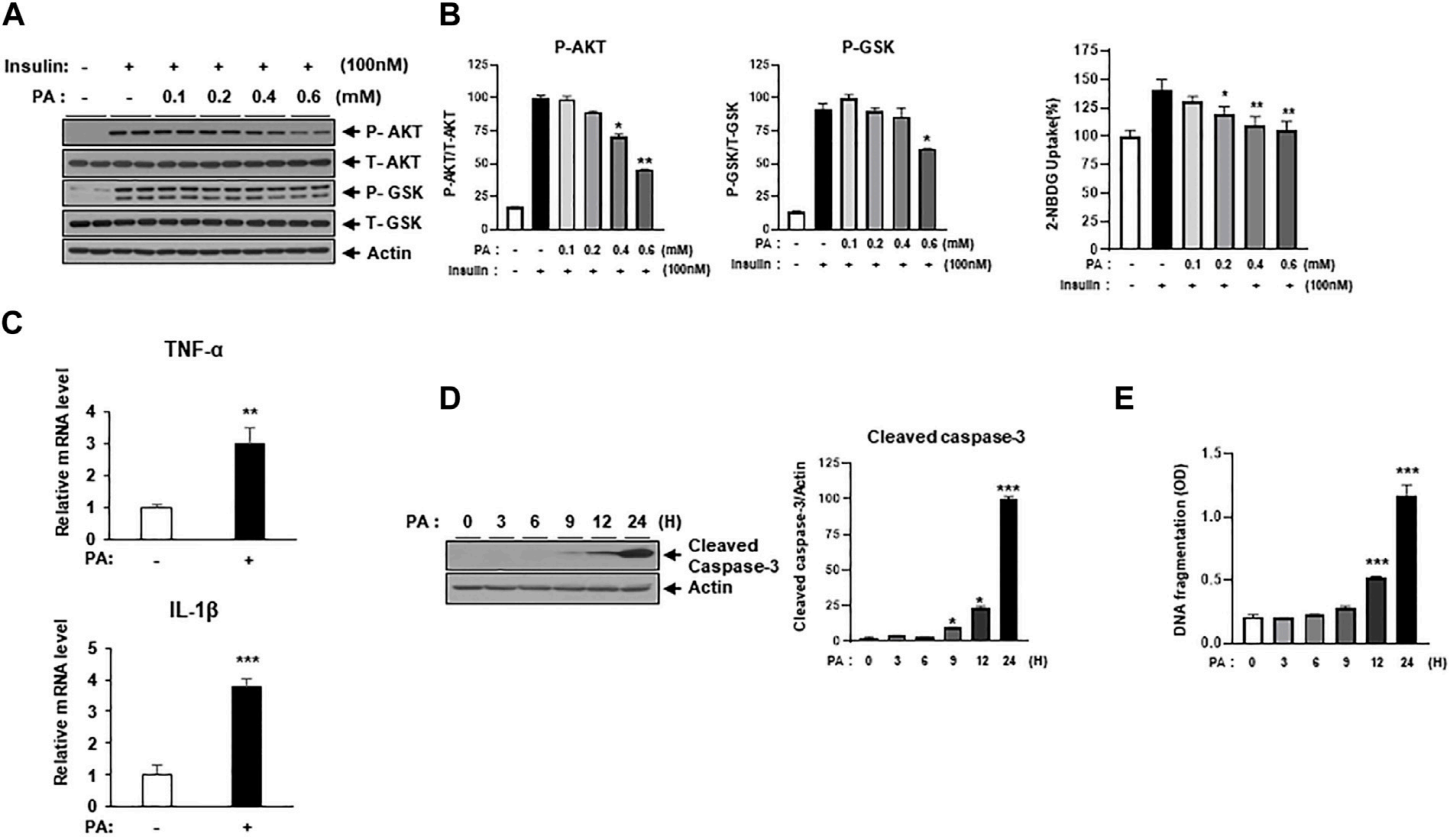
Palmitate (PA) induces lipotoxicity injury in H9c2 cells. **(A)** H9c2 cells were treated with different concentrations of PA for 8 h to induce insulin resistance. These cells were then starved for 4 h and treated with 100 nM of insulin for 30 min. Insulin resistance was assessed by immunoblotting using p-AKT and p-GSK antibodies. ^*^ *p* < 0.05; ^**^ *p* < 0.01 vs. p-AKT or p-GSK from insulin-treated H9c2 cells. **(B)** H9c2 cells were treated with different concentrations of PA for 8 h. These cells were then starved for 4 h and then treated with 500 μM 2-NBDG with or without 100 nM insulin at 37°C for 2 h. 2-NBDG uptake was measured with a fluorescence microplate reader. ^*^ *p* < 0.05; ^**^ *p* < 0.01 vs. insulin-treated H9c2 cells. **(C)** H9c2 cells were treated with 0.2 mM of PA for 8 h. Expression levels of inflammatory cytokines such as TNF-α and IL-1β were then measured using qRT-PCR. ^**^ *p* < 0.01; ^***^ *p* < 0.001 vs TNF-α or IL-1β from PA-untreated cells. **(D)** H9c2 cells were treated with 0.2 mM of PA for 24 h. Cleaved caspase-3 was then measured by immunoblotting using cleaved caspase-3 antibody. ^*^ *p* < 0.05; ^***^ *p* < 0.001 vs. cleaved caspase-3 from PA-untreated H9c2 cells. **(E)** H9c2 cells were treated with 0.2 mM of PA for indicated time periods. DNA fragmentation was then measured using a Cell Death Detection ELISA kit. Data are presented from three independent experiments. ^***^ *p* < 0.001 vs. fragmented DNA from PA-untreated H9c2 cells.

It is well-known that factors like hyperlipidemia, hyperglycemia, insulin resistance, high levels of AGEs, and lipotoxicity contribute to diabetic cardiomyocyte injury (Tan et al., 2020). To establish an *in vitro* system mimicking diabetic cardiomyocytes, H9c2 cells were treated with either high concentrations of glucose or PA, followed by assessments of insulin signaling and apoptotic signaling. In our system, long-term treatment with high glucose did not alter insulin signaling or levels of cleaved caspase-3, a representative apoptotic signal (Supplementary Figures S2A, B). However, PA treatment did result in cardiomyocyte injury, as evidenced by changes in insulin resistance, inflammatory gene expression, and cellular apoptotic signaling. H9c2 cells were treated with various concentrations of PA, followed by assessments of insulin signaling and glucose uptake. PA treatment decreased insulin-induced p-AKT and p-GSK-3α/β expression levels without changing total protein levels of Akt and GSK-3α/β (Figure 1A). Glucose uptake, measured via the 2-NBDG assay, showed that PA administration inhibited insulin-induced 2-NBDG uptake, implicating a decrease in glucose uptake via suppression of p-AKT and p-GSK-3α/β expression (Figure 1B).

Lipotoxicity also negatively impacts cardiomyocyte injury by increasing the expression of proinflammatory cytokines like TNF-α and IL1-β. These cytokines have been implicated in heart failure, affecting the phenotype and function of myocardial cells, contractile function in cardiomyocytes, and inflammatory activation in macrophages, among other roles (Hanna and Frangogiannis, 2020; Kaur et al., 2021). To examine whether PA elevates the expression of inflammatory cytokines, H9c2 cells were treated with 0.2 mM of PA for 8 h, followed by gene expression assessments for TNF-α and IL1-β (Figure 1C). PA treatment significantly increased the gene expression of both cytokines. Additionally, PA elevated levels of cleaved caspase-3 and DNA fragmentation, markers of cellular apoptosis (Figures 1D, E). These results suggest that PA treatment induces cardiomyocyte injury through impaired glucose uptake, upregulated inflammatory gene expression, and increased apoptotic signaling.

**FIGURE 2:**
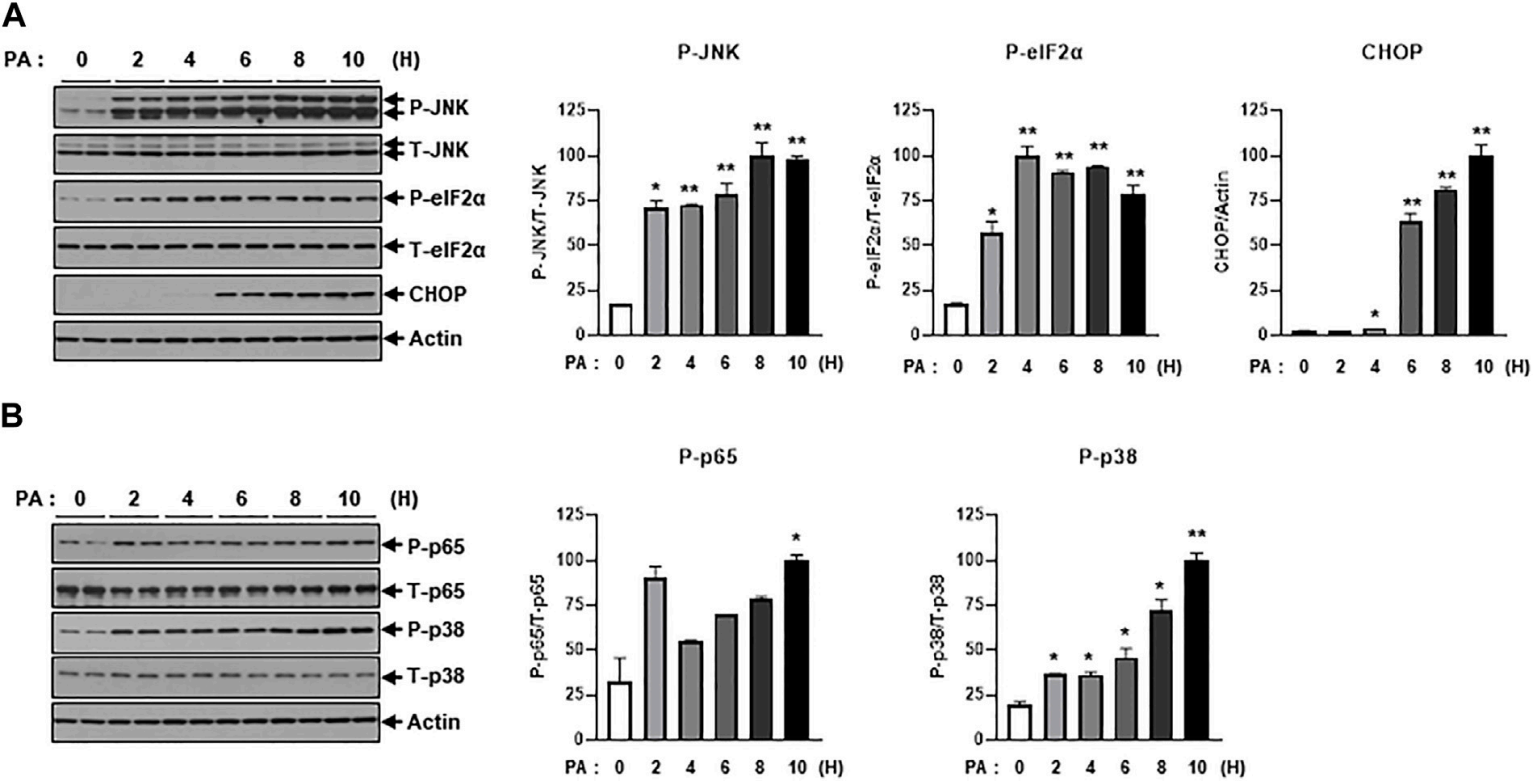
PA significantly increases ER stress and inflammation signaling. **(A)** H9c2 cells were treated with 0.2 mM of PA for indicated time periods. ER stress signaling molecules were then measured by immunoblotting using p-JNK, p-eIF2α, and CHOP antibodies. ^*^ *p* < 0.05; ^**^ *p* < 0.01 vs. phosphorylated molecules or CHOP from PA-untreated H9c2 cells. **(B)** Inflammatory signaling was detected by immunoblotting using p-p65 and p-p38 antibodies. ^*^ *p* < 0.05; ^**^ *p* < 0.01 vs. phosphorylated molecules from PA-untreated H9c2 cells.

Next, we investigated intracellular stress signals such as ER-stress and inflammation signaling under treatment of PA. Salvadó et al. reported that lipotoxicity induces insulin resistance via the activation of ER-stress signals such as p-IRE and p-JNK, which lead to the inhibition of IRS-1 phosphorylation (Salvadó et al., 2015). PA treatment increased ER-stress signals like p-JNK, p-eIF2α, and CHOP (Figure 2A). We also observed that PA administration stimulated inflammatory stress signals such as p-p65 and p-p38 (Figure 2B), which subsequently promoted the expression of proinflammatory cytokines like TNF-α and IL-1β (Figure 1C).

### 3.3 EMPA inhibits PA-induced insulin resistance, proinflammatory cytokine expression, and cellular apoptosis

**FIGURE 3:**
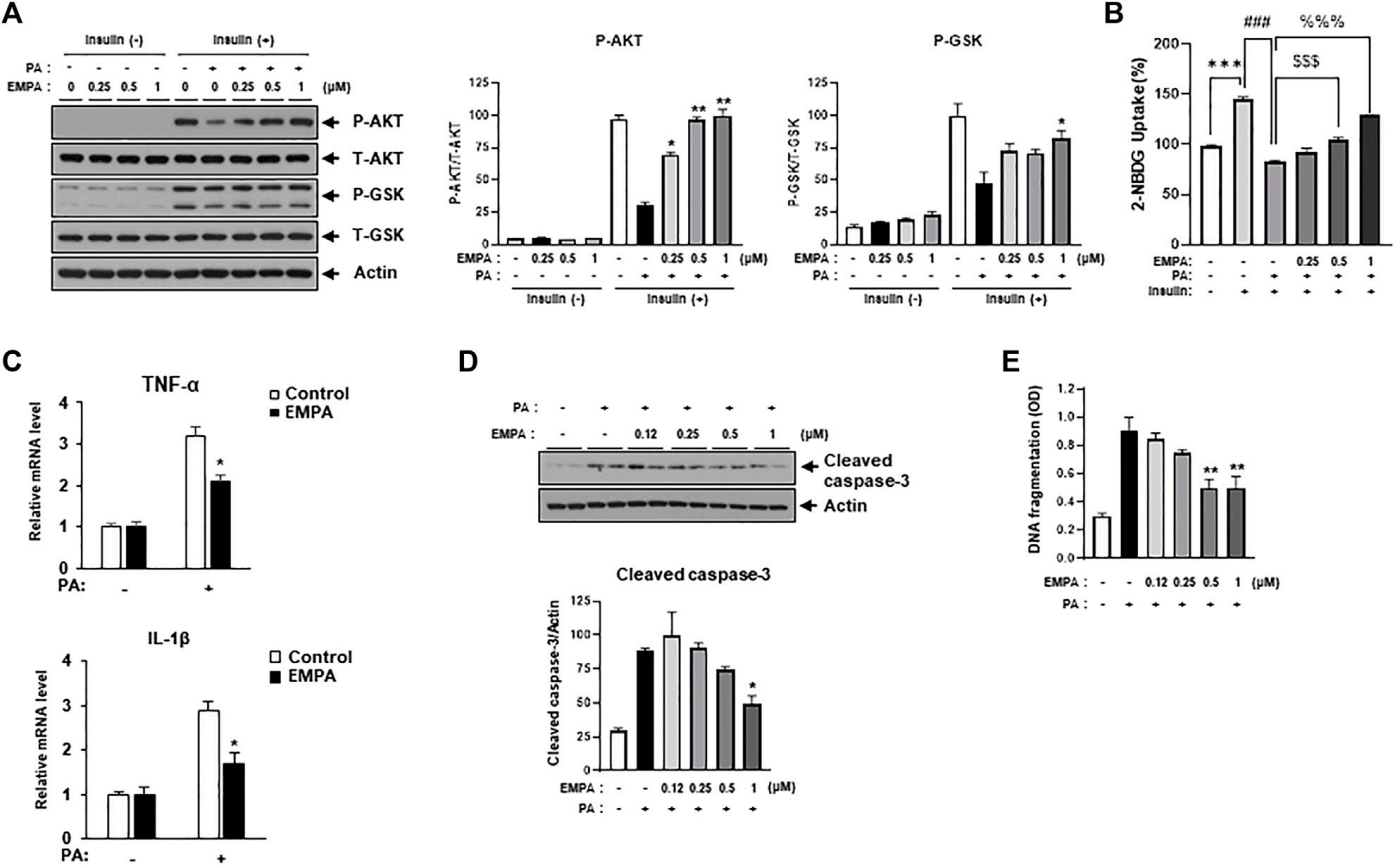
Empagliflozin (EMPA) protects H9c2 against PA-induced insulin resistance and apoptosis. **(A)** H9c2 cells were treated with 0.2 mM of PA with/without EMPA at indicated concentrations for 8 h. These cells were then starved for 4 h and treated with 100 nM of insulin for 30 min. Insulin resistance was assessed by immunoblotting using p-AKT and p-GSK antibodies. ^*^ *p* < 0.05; ^**^ *p* < 0.01 vs. p-AKT or p-GSK from PA-treated and insulin-treated H9c2 cells. **(B)** H9c2 cells were treated with 0.2 mM of PA with/without EMPA at indicated concentrations for 8 h. These cells were then starved for 4 h and then treated with 500 μM 2-NBDG with or without 100 nM insulin at 37°C for 2 h. 2-NBDG uptake was measured with a fluorescence microplate reader. ^***^ *p* < 0.001 vs. 2-NBDG uptake from untreated H9c2 cells; ^###^ *p* < 0.001 vs. 2-NBDG uptake from insulin-treated H9c2 cells; $$$ < 0.001 vs. 2-NBDG uptake from insulin- and PA-treated H9c2 cells; %%% < 0.001 vs. 2-NBDG uptake from insulin- and PA-treated H9c2 cells. **(C)** H9c2 cells were treated with 0.2 mM of PA with/without EMPA for 8 h. Expression levels of inflammatory cytokines such as TNF-α and IL-1β were then measured using qRT-PCR. ^*^ *p* < 0.05 vs. EMPA-untreated and PA-treated cells. **(D)** H9c2 cells were treated with 0.2 mM of PA with/without EMPA for 24 h and cleaved caspase-3 was measured by immunoblotting using cleaved caspase-3 antibody. ^*^ *p* < 0.05 vs. cleaved caspase-3 from EMPA-untreated cells. **(E)** H9c2 cells were treated with 0.2 mM of PA for 24 h at indicated concentrations of EMPA. DNA fragmentation was then measured using a Cell Death Detection ELISA kit. Data are presented from three independent experiments. ^**^ *p* < 0.01 vs. fragmented DNA from PA-treated and EMPA-untreated H9c2 cells.

**FIGURE 4:**
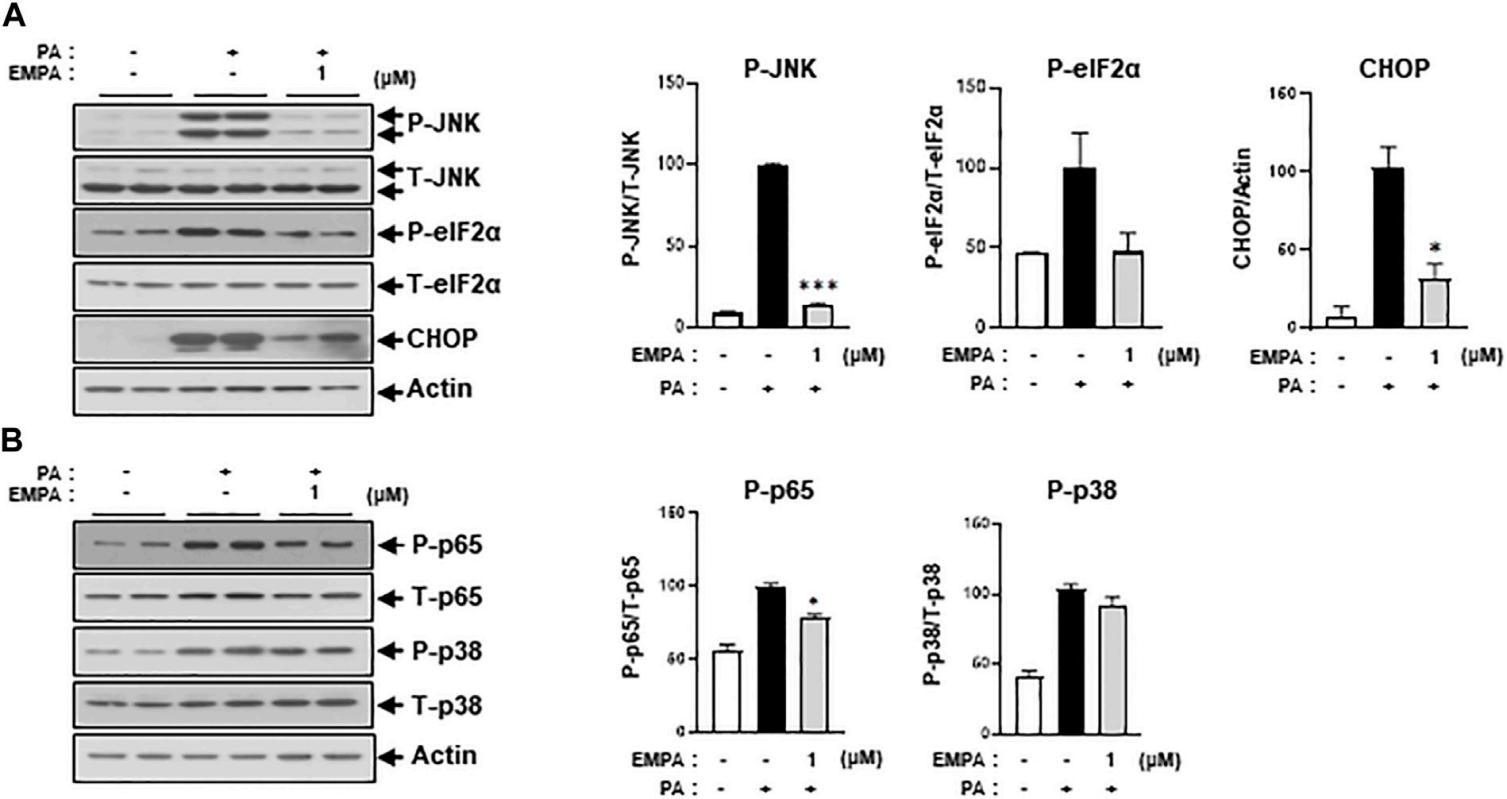
EMPA reduces ER stress and inflammatory signaling molecules. H9c2 cells were treated with 0.2 mM of PA with/without 1 μM of EMPA for 8 h **(A)** ER stress was detected by immunoblotting using p-JNK, p-eIF2α, and CHOP antibodies. ^*^ *p* < 0.05; ^***^ *p* < 0.001 vs. phosphorylated molecules or CHOP from PA-treated H9c2 cells. **(B)** Inflammatory signaling molecules were detected by immunoblotting using p-p65 and p-p38 antibodies. ^*^ *p* < 0.05 vs. phosphorylated molecules from PA-treated H9c2 cells.

We then sought to examine the effects of the SGLT2 inhibitor, EMPA, on PA-induced diabetic cardiomyocyte injury in H9c2 cells. EMPA treatment ameliorated the PA-induced reduction in p-AKT and p-GSK protein expression levels (Figure 3A), leading to improved 2-NBDG uptake in H9c2 cells (Figure 3B). These findings suggest that EMPA restores insulin-stimulated glucose uptake through upregulation of p-AKT expression, thereby increasing insulin sensitivity in H9c2 cells. EMPA also attenuated PA-induced expression of proinflammatory cytokines such as TNF-α and IL-β (Figure 3C). Moreover, PA-induced cleaved caspase-3 expression and DNA fragmentation were mitigated by EMPA (Figures 3D, E). We next explored whether EMPA could inhibit ER stress markers like p-JNK that affect insulin signaling. Treatment with 1 µM of EMPA reduced PA-induced ER stress signals including p-JNK, p-elF2, and CHOP expression (Figure 4A). EMPA also significantly lowered inflammation signals such as p-p65, which influence the production of proinflammatory cytokines like TNF-α and IL-β (Figure 4B), although it did not affect p-p38 levels. These results suggest that EMPA mitigates PA-induced cardiomyocyte injury, including insulin resistance, proinflammatory cytokine expression, and cellular apoptosis.

### 3.4 EMPA enhances CPT1 gene expression and lipid beta-oxidation

**FIGURE 5:**
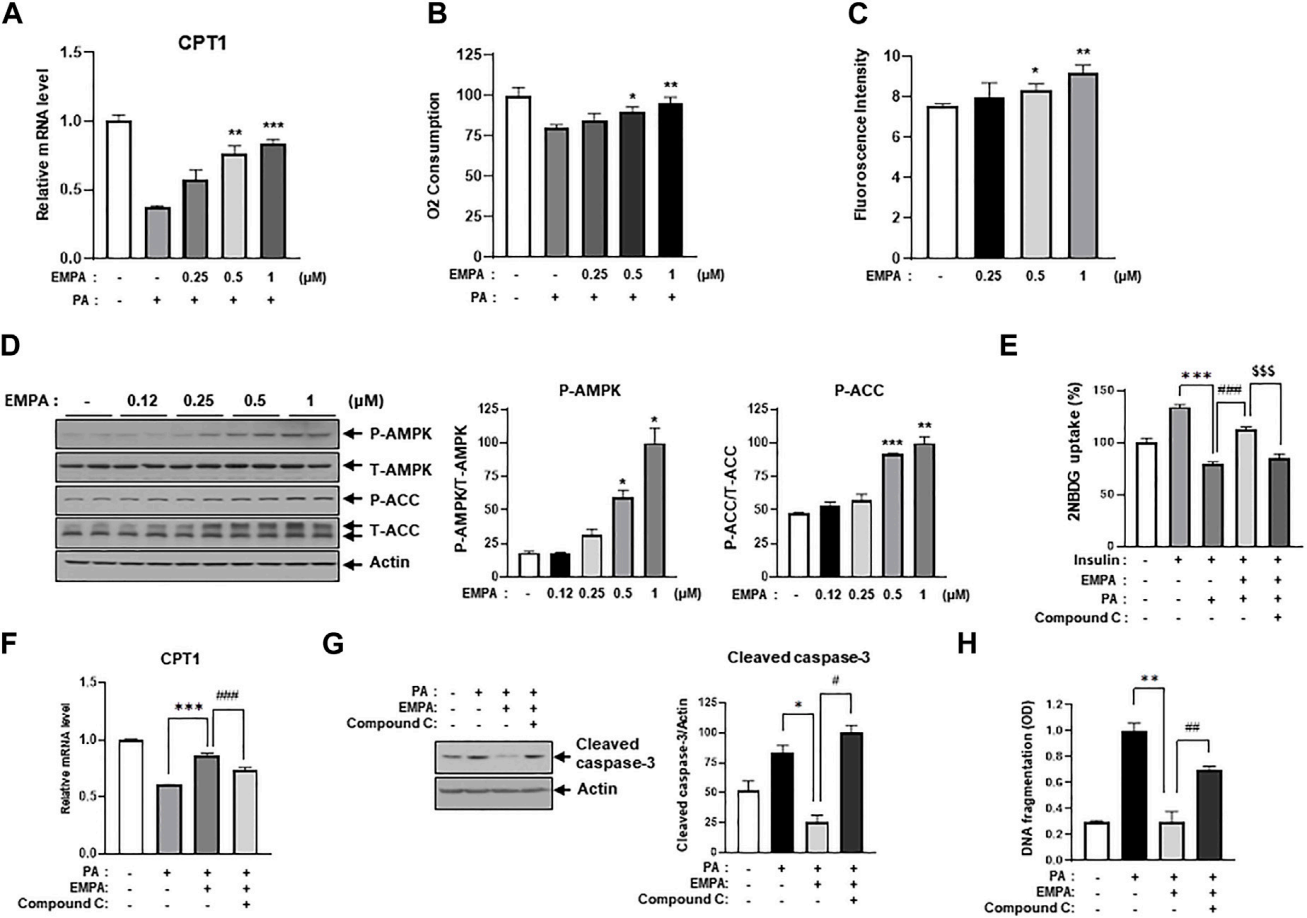
EMPA recovers PA-reduced β-oxidation through activation of AMPK and increased CPT1 gene expression. **(A)** H9c2 cells were treated with 0.2 mM of PA with/without EMPA at indicated concentrations for 8 h. CPT1 gene expression was then measured using qRT-PCR. ^**^ *p* < 0.01; ^***^ *p* < 0.001 vs. PA-treated cells. **(B)** H9c2 cells were treated with 0.2 mM of PA with/without EMPA at indicated concentrations for 8 h. Oxygen consumption rate (OCR) was then determined. ^*^ *p* < 0.05; ^**^ *p* < 0.01 vs. PA-treated and EMPA-untreated H9c2 cells. **(C)** H9c2 cells were treated with various concentrations of EMPA for 24 h and AMP level was measured using an AMP assay kit. ^*^ *p* < 0.05; ^**^ *p* < 0.01 vs. untreated H9c2 cells. **(D)** H9c2 cells were treated with various concentrations of EMPA for 24 h and the AMPK signaling pathway was detected by immunoblotting using p-AMPK and p-ACC antibodies. ^*^ *p* < 0.05; ^**^ *p* < 0.01; ^***^ *p* < 0.001vs. phosphorylated molecules from EMPA-untreated H9c2 cells. **(E)** H9c2 cells were treated with 0.2 mM of PA with/without EMPA or with/without compound C for 8 h. These cells were then starved for 4 h and then treated with 500 μM 2-NBDG with or without 100 nM insulin at 37°C for 2 h. 2-NBDG uptake was measured with a fluorescence microplate reader. ^***^ *p* < 0.001 vs. 2-NBDG uptake from insulin-treated H9c2 cells; ^$$$^ *p* < 0.001 vs. 2-NBDG uptake from PA-treated and insulin-treated H9c2 cells; ^*###*^ < 0.001 vs. 2-NBDG uptake from insulin-, EMPA-, and PA-treated H9c2 cells. **(F)** H9c2 cells were treated with 0.2 mM of PA with/without EMPA or with/without compound C for 8 h. CPT1 gene expression was then measured using qRT-PCR. ^*^ *p* < 0.05 vs. cleaved caspase-3 from PA-treated H9c2 cells; ^#^ *p* < 0.05 vs. cleaved caspase-3 from PA- and EMPA-treated H9c2 cells. **(G)** H9c2 cells were treated with 0.2 mM of PA with/without EMPA or with/without compound C for 24 h. Cellular apoptosis was assessed by immunoblotting using a cleaved-caspase3 antibody. ^*^ *p* < 0.05 vs. cleaved caspase-3 from PA-treated H9c2 cells; ^#^ *p* < 0.05 vs. cleaved caspase-3 from PA- and EMPA-treated H9c2 cells. **(H)** H9c2 cells were treated with 0.2 mM of PA with/without EMPA or with/without compound C for 24 h. DNA fragmentation was measured using a Cell Death Detection ELISA kit. ^*^ *p* < 0.05 vs. cleaved caspase-3 from PA-treated H9c2 cells; ^#^ *p* < 0.05 vs. cleaved caspase-3 from PA- and EMPA-treated H9c2 cells.

Since Figure 3 illustrated that EMPA ameliorates lipotoxic conditions such as insulin resistance and cellular apoptosis, we sought to identify the specific role of EMPA in cellular lipid metabolism. To this end, we assessed CPT1 gene expression, which is associated with lipid beta-oxidation, in the context of PA treatment with or without EMPA. EMPA restored the PA-induced decrease in CPT1 gene expression in a dose-dependent manner (Figure 5A), but not PDH gene expression or PDH activity (Supplementary Figures S3A, B). Intriguingly, EMPA alone also increased CPT1 gene expression (Supplementary Figure S3C). Subsequently, we measured the oxygen consumption rate (OCR), an indicator of lipid metabolism. PA-treated H9c2 cells exhibited a 75% reduction in OCR compared to untreated cells; however, EMPA significantly restored OCR in a dose-dependent manner (Figure 5B).

### 3.5 EMPA inhibits palmitate-evoked insulin resistance and cellular apoptosis through AMPK activation

We hypothesized that EMPA might induce AMPK activation and enhance mitochondrial metabolic activity, thereby increasing FFA oxidation. Interestingly, we found that EMPA elevates AMP levels in H9c2 cells in a dose-dependent manner (Figure 5C). Given that increased AMP levels can activate AMPK, we assessed the levels of activated AMPK and its upstream proteins through immunoblotting with p-AMPK and p-ACC antibodies. As anticipated, EMPA increased p-AMPK and p-ACC levels dose-dependently (Figure 5D). To understand which subunits are involved in AMPK activation by EMPA treatment, we assessed mRNA expression levels of AMPK subunits. As a result, the γ1 subunit of AMPK significantly increased when administered with EMPA (Supplementary Figure S4). These results suggest that EMPA has beneficial effects through stimulating AMP production and subsequently activating AMPK. To validate that the beneficial effect of EMPA is dependent on AMPK activation, we co-treated the cells with compound C, a specific AMPK inhibitor. We observed that EMPA significantly improved 2-NBDG uptake and CPT1 expression in PA-treated H9c2 cells, but co-treatment with compound C notably diminished these beneficial effects (Figures 5E, F). Similarly, EMPA also reduced palmitate-induced levels of cleaved caspase-3; however, co-treatment with compound C negated this positive effect (Figure 5G). Co-treatment with compound C also considerably inhibited EMPA’s protective effects against palmitate-induced DNA fragmentation (Figure 5H). These data support our hypothesis that AMPK plays a crucial role in the protective effects of EMPA against PA-induced injury in H9c2 cells.

## 4 Discussion

In this study, we demonstrated EMPA’s protective effects on excessive fatty acid-induced diabetic cardiomyocyte injury in H9c2 cells. We found that PA induced insulin resistance, pro-inflammatory cytokine production, and cellular apoptosis. However, co-treatment with EMPA ameliorated these injuries through activation of the AMPK pathway, which was linked to CPT1 gene expression and AMP production. This led to improved fatty acid oxidation and, ultimately, protected against diabetic cardiomyocyte injury.

The primary role of SGLT2i is to lower blood glucose levels through increased glucosuria. Numerous studies have highlighted the cardioprotective effects of SGLT2i in both animal models and patients. The underlying mechanisms of these inhibitors in patients with or without T2D are not fully understood but are likely to involve multiple factors, including non-glycemic benefits (Ferrannini et al., 2016; Kidokoro et al., 2019). To explore these alternative mechanisms, we treated H9c2 cells with PA *in vitro* and examined markers of cardiomyocyte injury like insulin resistance, pro-inflammatory cytokine production, and cellular apoptosis. Prior to the main study, we found that SGLT2 was expressed in various tissues, including the heart and H9c2 cells, as well as other metabolism-associated target organs such as the liver, muscles, and adipose tissue. This suggests that additional mechanisms for SGLT2i may exist in metabolic diseases.

AMPK is a master regulator of cellular energy metabolism (Hardie et al., 2016). It comprises two α, two β, and three γ subunits. The α subunits have a catalytic role, while the β and γ subunits have regulatory and targeting roles, respectively (Mitchelhill et al., 1997). Our study revealed that EMPA increased AMP levels and activated AMPK in H9c2 cells. Koyani et al. also reported that EMPA protects the heart from inflammation via AMPK activation in a lipopolysaccharide (LPS)-induced inflammation model, both *in vitro* and *in vivo* (Koyani et al., 2020). Similarly, Zhou H et al. found that EMPA reversed suppressed AMPK phosphorylation in diabetes (Zhou et al., 2018). These results suggest that the beneficial effects of EMPA are likely to stem from the activation of AMPK.

In diabetic conditions, long-term exposure to lipids diminishes fat oxidation and accumulates lipid droplets or intermediates, leading to insulin resistance. Our findings suggest that EMPA reverses PA-induced reductions in the OCR, which represents lipid oxidation and insulin sensitivity. Previous studies have shown that SGLT2 inhibitors improve insulin resistance by preserving mitochondrial fatty acid oxidation rates in the heart, muscle, and fat tissue under conditions of chronic lipid overload (Joannides et al., 2017; Makrecka-Kuka et al., 2020). Given that AMPK plays a vital role in fatty acid metabolism (Garcia and Shaw, 2017), reduced AMPK phosphorylation in diabetic insulin resistance may contribute to mitochondrial and cardiac dysfunction (Ye, 2013; Zhou et al., 2018). Hence, AMPK agonists have gained attention as a promising approach to improving insulin sensitivity (Zhang et al., 2009). Sun et al. reported that EMPA treatment decreased lipid content in HFD hearts through activation of ACC phosphorylation, leading to enhanced fatty acid oxidation (Sun et al., 2020). We found similar results; EMPA restored PA-induced downregulation of OCR and increased levels of phosphorylated AMPK and ACC.

Additionally, we found that EMPA treatment alone increased CPT1 gene expression, which is located in mitochondrial membranes and plays a key role in the formation of acylcarnitines essential for fatty acid β-oxidation. EMPA also rescued the PA-induced reduction of CPT1 gene expression. This phenomenon is likely to play a vital role in enhancing fatty acid oxidation. Therefore, we hypothesize that EMPA protects mitochondria from lipid overload-induced damage through enhanced fatty acid oxidation, facilitated by the activation of CPT1 and AMPK. Ultimately, EMPA helps restore insulin resistance, reduces inflammatory cytokine gene expression, and decreases cellular apoptosis in H9c2 cells. Our data provide insights into the potential of EMPA as a novel therapeutic strategy, not only for antidiabetic conditions but also for metabolic diseases, particularly cardiomyopathy in tissues expressing SGLT2.

## Data Availability

The original contributions presented in the study are included in the article/Supplementary Material, further inquiries can be directed to the corresponding author.

## Abbreviations

SGLT2, Sodium-glucose cotransporter 2; EMPA, Empagliflozin; PA, palmitate; CPT1, Carnitine palmitoyl transferase1; FFA, Free fatty acid; ROS, Reactive oxygen species; BSA, Bovine serum albumin; PBS, phosphate-buffered saline; ROS, Reactive oxygen species; OCR, Oxygen consumption rate; 2-NBDG, 2-[N-(7-Nitrobenz-2-oxa-1,3 diazol-4-yl) amino]-2-deoxy-D-glucose (2-NBDG); FAO, Fatty acid oxidation; AMPK, AMP-activated kinase; CHOP, C/EBP Homologous Protein; ER, endoplasmic reticulum; eIF2α, eukaryotic translation initiation factor 2α; JNK, c-Jun N-terminal kinase 1; GSK3 α/β, Glycogen synthase kinase 3 α/β; NF-κB, nuclear factor-κB.
