# Supplementary figures and images for: Protective effect of empagliflozin against palmitate-induced lipotoxicity through AMPK in H9c2 cells
Source: Front Pharmacol. 2023 Dec 5;14:1228646. doi: 10.3389/fphar.2023.1228646 (PMC10728651; doi:10.3389/fphar.2023.1228646)

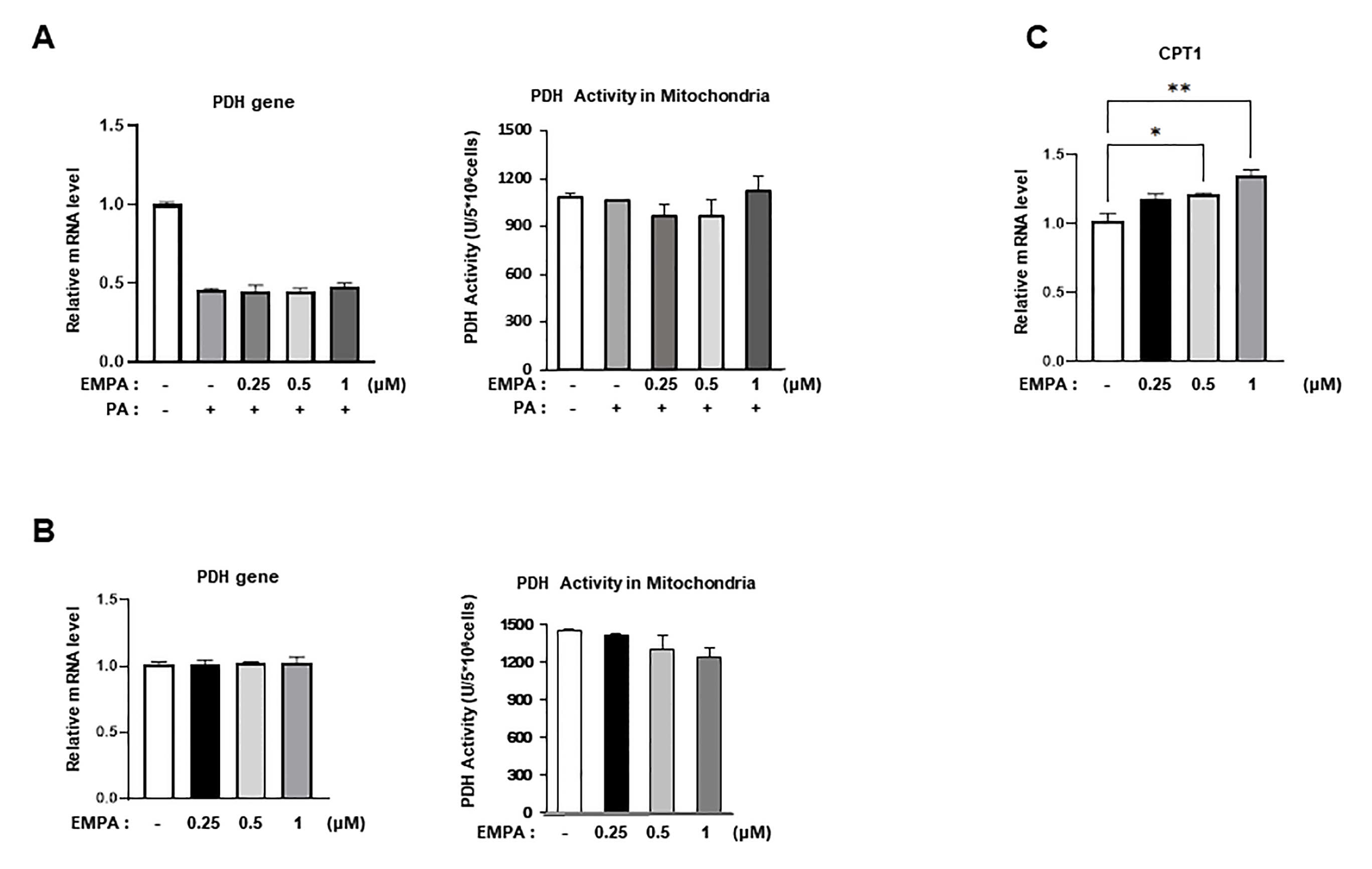

Supplement: Supplementary file 1 [file Image3.TIF]

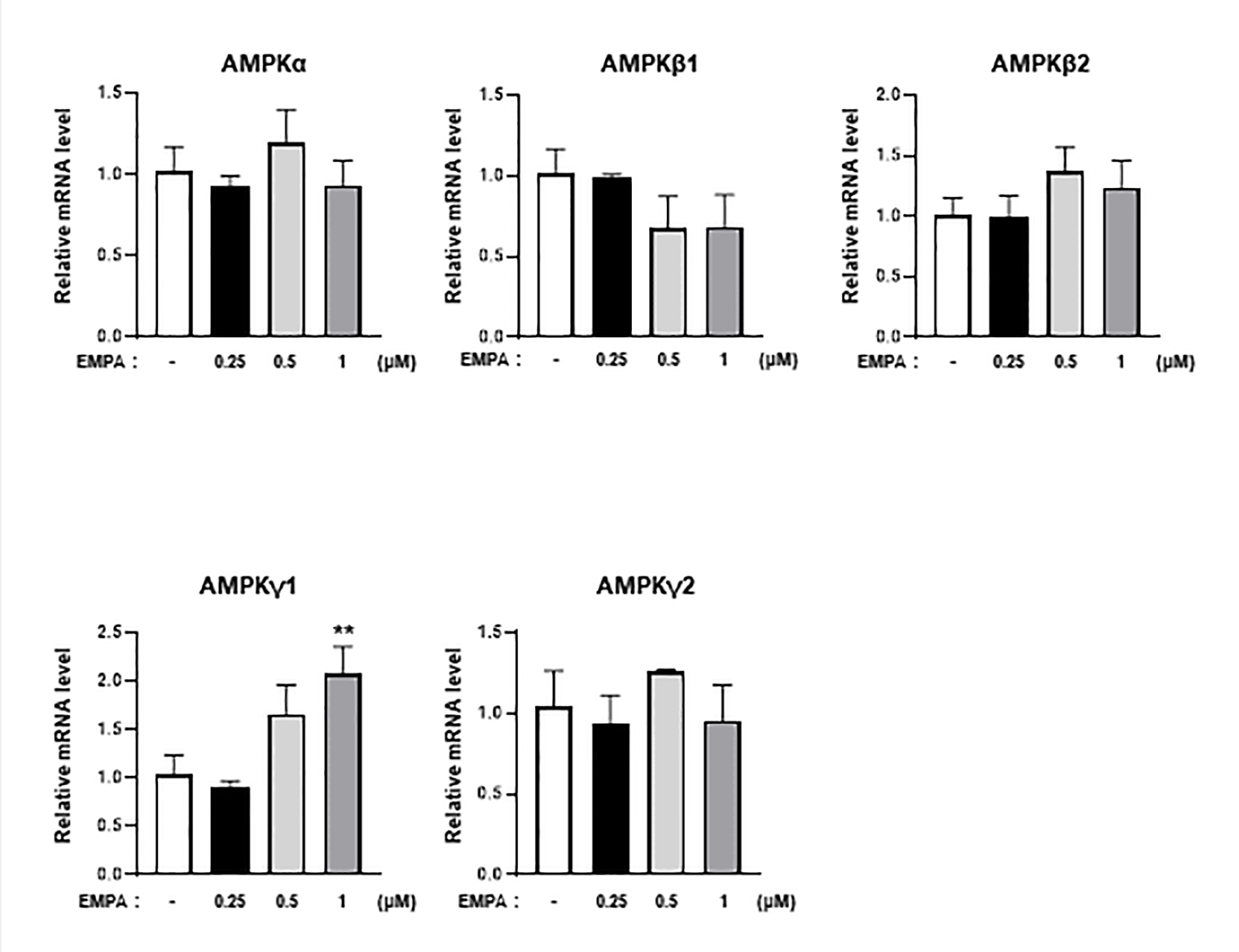

Supplement: Supplementary file 2 [file Image4.TIF]

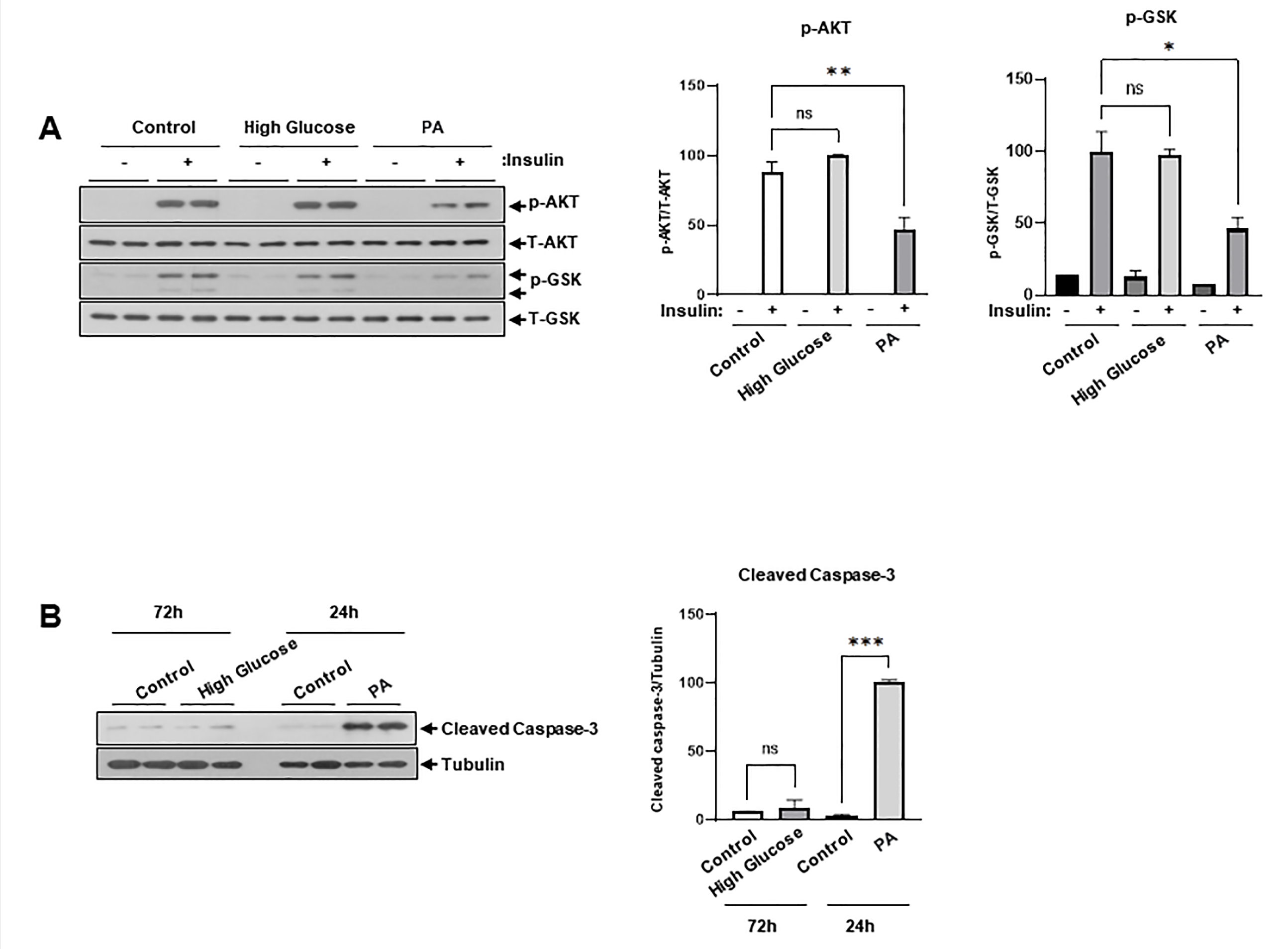

Supplement: Supplementary file 3 [file Image2.TIF]

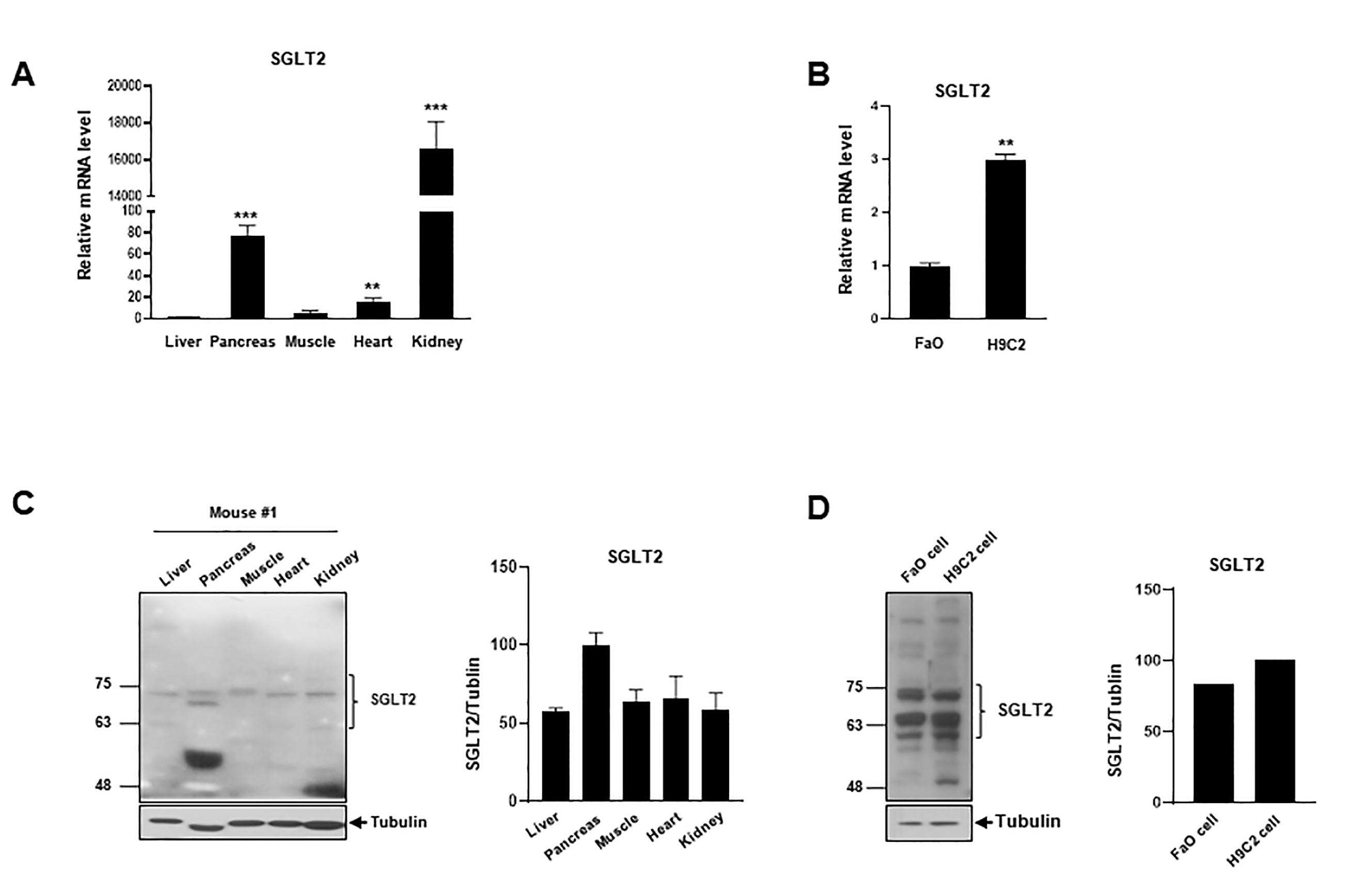

Supplement: Supplementary file 4 [file Image1.TIF]
