# Supplementary material for: Protective effect of empagliflozin against palmitate-induced lipotoxicity through AMPK in H9c2 cells
Source: Front Pharmacol. 2023 Dec 5;14:1228646. doi: 10.3389/fphar.2023.1228646 (PMC10728651; doi:10.3389/fphar.2023.1228646)
Supplement: Supplementary file 5 [file DataSheet1.pdf]

Supplementary Material

Supplementary Figures

Supplementary Figure 1. SGLT2 expression levels in several rat tissues. RNAs were isolated from several rat tissues. SGLT2 expression level was measured by qRT-PCR using specific primers (A). RNAs were isolated from rat cell lines (FaO and H9c2) and SGLT2 expression level was measured by qRT-PCR (B). $^{**}p < 0.01$; $^{***}p < 0.001$ vs. SGLT2 level from liver tissue. SGLT2 protein levels in several rat tissues were determined. Proteins were isolated from rat tissues. SGLT2 protein level was measured by immunoblotting using SGLT2 antibody (C). Proteins were isolated from rat cell lines (FAO, and H9c2). SGLT2 protein level was then measured by immunoblotting using SGLT2 antibody (D).

Supplementary Figure 2. Palmitate (PA) induces lipotoxicity in H9c2 cells.

(A) H9c2 cells were treated with PA for 8 hrs or high glucose (25mM) for 72 hrs. These cells were then starved 4 hrs and treated with 100 nM of insulin for 30 min. Insulin resistance was assessed by immunoblotting using p-AKT and p-GSK antibodies. $^{*}P < 0.05$; $^{**}P < 0.01$ vs. p-AKT or p-GSK from insulin-treated H9c2 cells. (B) H9c2 cells were treated with PA for 24 hrs or high glucose (25mM) for 72 hrs. Cellular apoptosis was assessed by immunoblotting using cleaved-caspase3 antibody. $^{***}p < 0.001$ vs. cleaved-caspase 3 from PA-untreated H9c2 cells.

Supplementary Figure 3. EMPA does not change expression or activity of PDH, which is related to glucose oxidation. (A) H9c2 cells were treated 0.2 mM of PA with/without EMPA at indicated concentrations for 8 hrs. PDH gene expression level and PDH activity were then measured using qRT-PCR and PDH activity assay kit, respectively. (B) H9c2 cells were treated with or without

EMPA at indicated concentrations for 24 hrs. PDH gene expression and PDH activity were then measured using qRT-PCR and PDH activity assay kit, respectively. (C) CPT1 gene expression was measured using qRT-PCR.

Supplementary Figure 4. Treatment of H9c2 with EMPA increases expression of AMPKγ1 subunit. H9c2 cells were treated with various concentrations of EMPA for 24 h. AMPK subunit expression level was measured by qRT-PCR using specific primers. $^{**}p < 0.01$ vs. expression of AMPK γ1 subunit from EMPA-untreated H9c2 cells.

Supplementary Table 1. List of primers used in this study.

**Name**	**Sequence**	**Supplier**
Rat IL-1β (Forward primer)	5’-GTC ACT CAT TGT GGC TGT GG-3’	Bioneer, Korea
Rat IL-1β (Reverse primer)	5’-AAA GAA GGT GCT TGG GTC CT-3’	Bioneer, Korea
Rat TNF-α (Forward primer)	5’-ATG TGG AAC TGG CAG AGG AG-3’	Bioneer, Korea
Rat TNF-α (Reverse primer)	5’-CGA GCA GGA ATG AGA AGA GG-3’	Bioneer, Korea
Rat RPL32 (Forward primer)	5’-TCC ACA ATG TCA AGG AGC TG-3’	Bioneer, Korea
Rat RPL32 (Reverse primer)	5’-CGA TGG CTT TTC GGT TCT TA-3’	Bioneer, Korea
Rat SGLT2 (Forward primer)	5’-CTG AAC TTG GGG AGC AGA AG-3’	Bioneer, Korea
Rat SGLT2 (Reverse primer)	5’-CAC AAG CCA ACA CCA ATG AC-3’	Bioneer, Korea
Rat AMPKα1 (Forward primer)	5’-TGG CTT CGT TCA TTA TTC TCC T-3’	Bioneer, Korea
Rat AMPKα1 (Reverse primer)	5’-CAG CTG CCT GTA CAA CAG AAA C-3’	Bioneer, Korea
Rat AMPK β1 (Forward primer)	5’-CTA CCA CCA GGA GCC TTA CAT C-3’	Bioneer, Korea
Rat AMPK β1 (Reverse primer)	5’-TGT CCT TGT TCA AGA TGA CCT G-3’	Bioneer, Korea
Rat AMPK β2 (Forward primer)	5’-TGT CCA AGG GTC AGA GTG TG-3’	Bioneer, Korea
Rat AMPK β2 (Reverse primer)	5’-ATC AAC CCA TGC AAG GAA AG-3’	Bioneer, Korea

Rat AMPK γ1 (Forward primer)	5'-GGA GAG AGG TCT ACC TGC AAG-3'	Bioneer, Korea
Rat AMPK γ1 (Reverse primer)	5'-TGG AAG CCT GTG GAT CTT ATT T-3'	Bioneer, Korea
Rat AMPK γ2 (Forward primer)	5'-CTG TCA GAC ATC CTG CAA GC-3'	Bioneer, Korea
Rat AMPK γ2 (Reverse primer)	5'-TGT GAG GGC GTC TAC ACT TG-3'	Bioneer, Korea
Rat CPT1 (Forward primer)	5'-TAT GTG AGG ATG CTG CTT CC-3'	Bioneer, Korea
Rat CPT1 (Reverse primer)	5'-CTC GGA GAG CTA AGC TTG TC-3'	Bioneer, Korea
Rat PDH (Forward primer)	5'-GGA TCA ATG CAC ATG TAC GC-3'	Bioneer, Korea
Rat PDH (Reverse primer)	5'-TAT ACT TGC AGG CCA GAG CA-3'	Bioneer, Korea
